# The Acute and Chronic Effects of Lion’s Mane Mushroom Supplementation on Cognitive Function, Stress and Mood in Young Adults: A Double-Blind, Parallel Groups, Pilot Study

**DOI:** 10.3390/nu15224842

**Published:** 2023-11-20

**Authors:** Sarah Docherty, Faye L. Doughty, Ellen F. Smith

**Affiliations:** 1 Department of Psychology, Northumbria University, Newcastle upon Tyne NE1 8ST, UK; 2 Brain, Performance and Nutrition Research Centre, Department of Psychology, Northumbria University, Newcastle upon Tyne NE1 8ST, UK; ellen.f.smith@northumbria.ac.uk

**Keywords:** cognitive function, mood, mushroom, *Hericium erinaceus*

## Abstract

Background: Given the bioactive properties and limited work to date, *Hericium erinaceus* (Lion’s mane) shows promise in improving cognitive function and mood. However, much of the human research has concentrated on chronic supplementation in cognitively compromised cohorts. Objective: The current pilot study investigated the acute and chronic (28-day) cognitive and mood-enhancing effects of *Hericium erinaceus* in a healthy, young adult cohort. Design: This randomized, double-blind, placebo-controlled, parallel-groups design investigated the acute (60 min post dose) and chronic (28-day intervention) effects of 1.8 g *Hericium erinaceus* in 41 healthy adults aged 18–45 years. Results: Analysis revealed that following a single dose of *Hericium erinaceus*, participants performed quicker on the Stroop task (*p* = 0.005) at 60 min post dose. A trend towards reduced subjective stress was observed following 28-day supplementation (*p* = 0.051). Conclusions: The findings tentatively suggest that *Hericium erinaceus* may improve speed of performance and reduce subjective stress in healthy, young adults. However, null and limited negative findings were also observed. Given the small sample size, these findings should be interpreted with caution. Further investigation in larger sample sizes is crucial, however the findings of this trial offer a promising avenue of interest.

## 1. Introduction

*Hericium erinaceus* (lion’s mane) is an edible mushroom, that belongs to the Hericiaceae family, order Russulales, class Agaricomycete and phylum Basidiomycota [1,2]. It is extensively found in East Asian countries including Japan and China [3]. The mature mushroom is easily identifiable, consisting of a number of single, long, dangling fleshy spines which are white in colour [4]. *H. erinaceus* has traditionally been used as a herbal medicine in East Asian countries with well documented health-promoting effects [5,6].

The medicinal properties of *H. erinaceus* include antioxidant, antimicrobial and anticancer effects [7]. Recently, interest has been placed on the potential neuroprotective and neuroregenerative properties of *H. erinaceus* [8,9]. These are likely underpinned by the numerous bioactive components identified within the mycelia and fruiting bodies of *H. erinaceus*, including polysaccharides, phenolic acids and terpenoids, specifically hericenones and erinacines [6,9]. Interestingly, both hericenones and erinacines can easily cross the blood–brain barrier [10]; promote nerve growth factor synthesis and secretion [11,12]; and, in animal models, have been shown to exert anti-neuroinflammatory and neuroprotective properties [4,13,14,15] and promising cognition-enhancing effects [16,17]. Additionally, an increase in circulating pro-brain-derived neurotrophic factor (BDNF) has been observed following hericene A (isolated from *H. erinaceus*) administration in mice [18]. The same effect was observed, alongside memory and mood enhancements, in overweight humans following *H. erinaceus* extract supplementation [19], suggesting a general effect on BDNF synthesis and a BDNF-like neurotrophic effect of *H. erinaceus* and its derivatives. Despite promising results from preclinical trials, limited research has investigated the potential cognition-enhancing effects of *H. erinaceus* in humans, with much of this work focusing on cognitively compromised demographics.

Preliminary research observed improvements in scores of mild cognitive impairment in 50- to 80-year old adults following 16 weeks of 3 g/day *H. erinaceus* supplementation [20]. This improvement was observed throughout the trial period at assessment during weeks 8, 12 and 16, but not at the follow-up observation 4 weeks after treatment cessation, suggesting that prolonged supplementation may be necessary to exert beneficial effects. Similar reductions in cognitive decline were observed in patients diagnosed with mild Alzheimer’s disease following 49-week supplementation with erinacine A-enriched *H. erinaceus* (three 350 mg capsules daily, each containing 5 mg/g erinacine A) [21]. This trial observed improvements on the Mini-Mental State Examination at week 49, alongside significant treatment-related differences in Instrumental Activities of Daily Living scores. When taken together, this suggests better cognition and a lower level of dependence following *H. erinaceus* supplementation. Whilst these trials provide promise in the potential for chronic supplementation of *H. erinaceus* to improve cognitive impairment in older adults with diagnosed cognitive impairment, it is important to understand if *H. erinaceus* could be employed before cognitive impairment and exert similar, cognition-enhancing effects in healthy, cognitively intact individuals.

To date, studies in this demographic are limited and often target healthy, older adults or assess mood effects in isolation. Here, improved scores on the Mini-Mental State Examination have been observed in adults aged ≥50 years who consumed 3.2 g of powdered fruiting body daily for 12 weeks [22], although no additional effects were observed on other measures. Most recently, a trial in a college-age athlete cohort aimed to assess the cognitive effects of 4-week *H. erinaceus* supplementation (10 g/day, presented in muffin form) [23]. Their findings indicated no effect on a dual-task challenge, comprising the Stroop task followed by a mental arithmetic task whilst simultaneously completing a Y-balance test. Although the dual-task challenge used has been well validated as a stressor and inducer of fatigue [24,25], it is limited in the cognitive domains it measures. Given the limited evidence of *H. erinaceus* on cognition, it would be beneficial to assess a wider range of cognitive domains to identify potential avenues of interest for supplementary research.

Additional trials suggest a mood-enhancing effect of *H. erinaceus*, with lowered depression and anxiety scores observed in menopausal women following 4 weeks of supplementation with *H. erinaceus*-containing cookies (four consumed each day, each with 0.5 g of powdered fruiting body) [26]. Similar reductions in depression, anxiety and sleep disorder scores were observed in overweight and obese adults following 8-week 550 mg *H. erinaceus* (80% bulk mycelia, 20% fruiting body extract) supplementation in combination with a low-calorie diet [19]. These mood improvements were associated with a change in peripheral pro-BNDF.

Given the current evidence, lion’s mane mushrooms may have the potential to elicit both cognitive and mood effects through various biological pathways. However, it is important to note that the area is within its infancy, with limited placebo-controlled, double-blind studies testing these effects. The trials to date are limited in terms of investigating single areas of brain function, such as markers of cognitive decline or specific aspects of mood, and use varying doses of *H. erinaceus* which are presented in differing forms. Moreover, each are presented over a chronic intervention period (4 to 49 weeks) with no work to date assessing the potential acute effects of *H. erinaceus* supplementation. And with the exception of one recent trial [23], all research to date has been conducted on either older adult samples, or specific clinical samples, typically in models of cognitive decline.

To further understand the potential cognitive and mood-enhancing effects of *H. erinaceus*, it is important to consider other demographic groups. Of particular interest are younger, healthy adults without cognitive decline. Given the potential neuroprotective properties, it is important to assess if *H. erinaceus* can exert beneficial effects prior to cognitive decline, with the potential to be used as a natural preventative measure in this population in the future.

Based on this the current pilot study had two main aims:

(1) To investigate the acute effects of lion’s mane on cognitive performance in healthy, young adults.
(2) To investigate the chronic effects of four-week supplementation of lion’s mane in healthy, young adults, with the primary outcome being accuracy and reaction time on global cognitive performance.

## 2. Materials and Methods

### 2.1. Study Design and Participants

The research trial employed a randomised, placebo-controlled, double-blind, parallel-groups design with two treatment groups. Data collection occurred between April and July 2023 at the Brain, Performance and Nutrition Research Centre (BPNRC), situated within Northumbria University. Ethical approval was provided by the Northumbria University Health and Life Sciences Ethics Committee (Reference 3528), with adherence to the principles outlined in the Declaration of Helsinki 1975.

**Figure 1:**
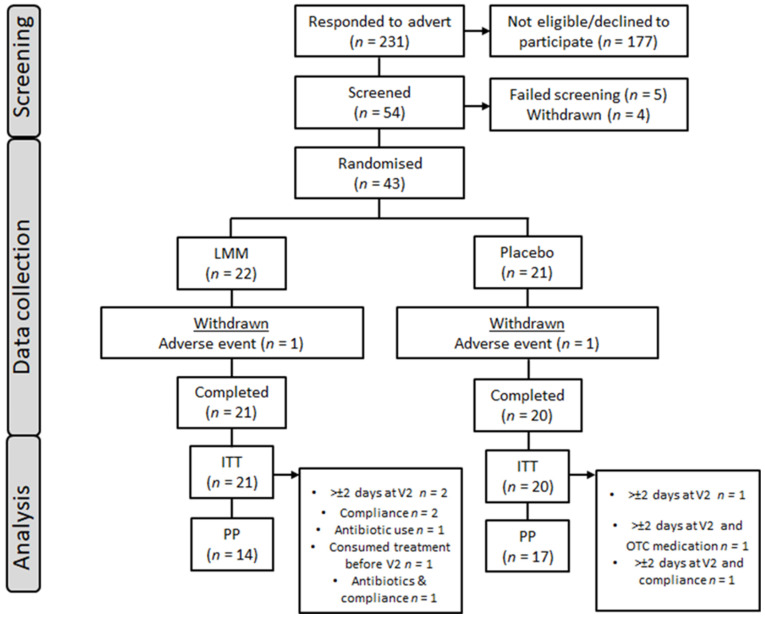
Participant disposition flowchart.

A sample of 43 volunteers aged 18–45 years (with a mean age 26.35 years) was recruited and randomised into the trial. Two participants discontinued their participation after the initial testing visit, and they were subsequently replaced. The data from the first testing visit of the discontinued participants were retained and included in the final analysis, resulting in a total sample of 43 participants for the acute analysis within Day 1 and 41 participants for the chronic intervention analysis. For an overview of participant enrolment and flow, see Figure 1. Participants indicated that they were in good health, without chronic disease and with a BMI within the 18.5–35 kg/m^2^ range. Further information regarding the inclusion and exclusion criteria is detailed within the Supplementary Materials. Physiological measurements (including blood pressure, height, weight, and waist-to-hip ratio (WHR)) were conducted during the participants’ introductory laboratory visit, while the participants themselves reported all other exclusion criteria. Detailed participant characteristics at the time of enrolment are available in the Supplementary Materials.

It has previously been recommended that a pilot study recruits between 12 and 30 participants per group [27,28]. Given this, the current study aimed to recruit 40 participants, with 20 participants in each treatment group and an even split of male and females in each treatment condition. The aim of this pilot trial was to support the concept of assessing the acute and chronic effects of *Hericium erinaceus* in healthy, young adults and, specifically, to inform future projects of potential areas of cognition and mood with the most promise.

### 2.2. Treatments

Participants were assigned to their treatment groups in a double-blind manner using a computer-generated randomization sequence. The treatment regimen consisted of taking three white vegetarian capsules daily, with each capsule containing either placebo (microcrystalline cellulose) or 600 mg of lion’s mane mushroom (SO-DSX1^®^, Sempera Organics Inc., Morgan Hill, CA, USA) (equating to a daily dose of 1.8 g). Each lion’s mane capsule contained a proprietary blend of organic *Hericium erinaceus* mushroom complex.

Participants were given a single bottle and were instructed to take three capsules every morning after having breakfast. The first and last doses of their 28-day treatment plan were administered under laboratory supervision, while the intermediate doses were self-administered at home. Adherence to the treatment was monitored through daily completion of a treatment diary, and a pill count was conducted during the final laboratory visit, revealing an average compliance of 96%. Participants reported any adverse health outcomes to assess tolerability and side effects associated with the intervention.

### 2.3. Cognitive and Mood Assessments

Cognitive function tests were delivered via the Computerised Mental Performance Assessment System (COMPASS), developed by Northumbria University (Newcastle upon Tyne, UK). COMPASS enables the creation of customised task configurations, with randomised parallel versions of these tasks administered to each participant during each assessment.

**Figure 2:**
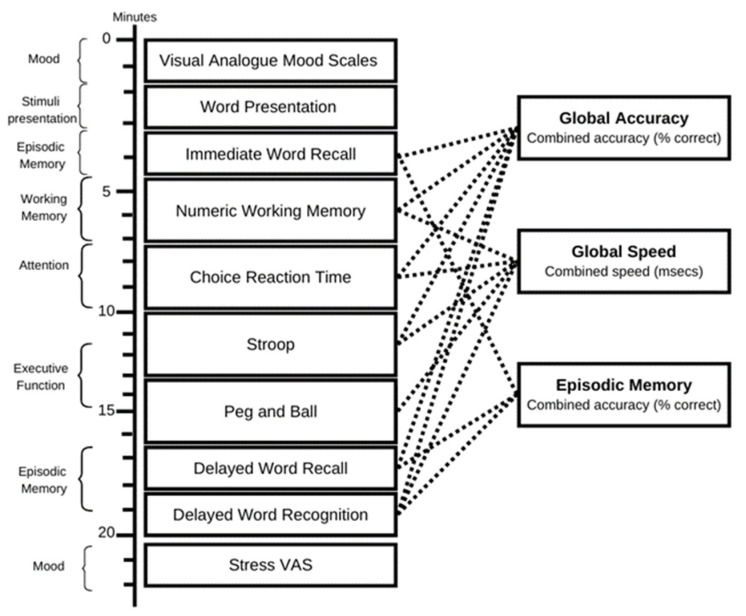
Cognitive assessments. The running order of tasks and their contribution to the cognitive factors (to the right). The battery of tasks took approximately 25 min to complete. This identical assessment was carried out both at the pre-treatment baseline and 60 min after dosage on both Day 1 and Day 29.

Given the novelty of the area and to provide insight on the future direction of the research, the configuration of cognitive tasks was developed to explore the effects of lion’s mane on a range of cognitive domains. Therefore, to measure effects on attention, executive function, working memory and episodic memory, the following tasks were employed: immediate and delayed word recall; numeric working memory; choice reaction time; Stroop task; peg and ball; delayed word recognition. These tasks, administered via COMPASS, have been demonstrated to be responsive to nutritional interventions [29,30,31,32,33]. The timelines for each assessment are depicted in Figure 2, while comprehensive task descriptions are available in the Supplementary Materials.

Mood and psychological state were assessed with the Stress Visual Analogue Scales (S-VAS), Visual Analogue Mood Scales (VAMS) and Perceived Stress Scale (PSS) [34]. The VAMS were created and validated internally. These scales consist of 18 lines anchored at opposing antonyms (e.g., ‘alert’ and ‘drowsy’). Participants provide responses based on their current feelings by marking a point along the line between the two words, and the ratings are scored from left to right. Responses from the 18 individual scales are then condensed into three composite scores, namely ‘alertness’, ‘stress’ and ‘tranquillity’, based on the results of a factor analysis. The S-VAS comprises four scales, each anchored with ‘not at all’ and ‘extremely’ at either side, and asks participants to rate their current levels of anxiety, stress, relaxation and calmness. By reversing the relaxed and calm scores, the scores from these four scales can be combined to generate an average ‘stress’ score. The PSS is a well-established measure of the perception of stress that has been validated in numerous populations. It requires participants to complete a 10-item questionnaire using a 5-point scale, based on their feelings within the previous month. A total score is calculated by reversing the positively stated items and summing the responses.

### 2.4. Procedure

Participants attended four appointments as part of the study protocol: a screening appointment, a training visit and two testing visits. The remote screening session was conducted via telephone and encompassed several key components. These included an explanation of the trial requirements, signing of an electronic consent form, collection of demographic information, review of the self-reported health screening encompassing the inclusion and exclusion criteria and calculation of caffeine intake (via the Caffeine Consumption Questionnaire (CCQ)). Following the telephone screening, the participants who met the eligibility criteria proceeded to attend a training visit, during which they signed a paper consent form. Physiological data, which could not be gathered remotely, including blood pressure, height, weight and waist-to-hip ratio (WHR) were also collected at this visit. Participants received training on the cognitive tasks and mood assessment scales, ensuring their understanding of the task instructions and test procedures. This training assessment also allowed for identification and exclusion of participants who were unable or unwilling to perform at a minimum standard.

**Figure 3:**
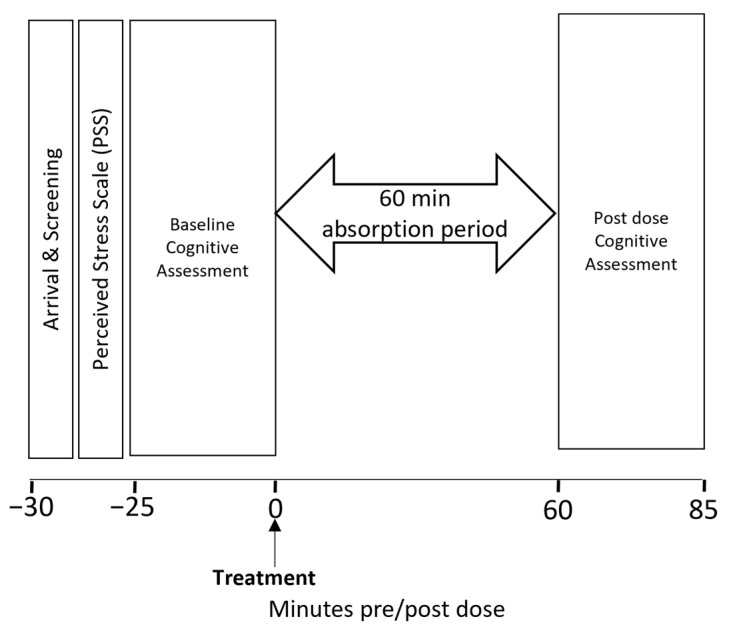
Timeline of the testing visit assessment schedule.

For the two subsequent laboratory-based testing sessions (referred to as Day 1 and Day 29), the participants arrived at the laboratory at either 8 am or 10 am (consistent between visits). Prior to their arrival, participants consumed a standardised breakfast of cereal or toast, ensuring that it was no later than an hour before their scheduled appointment. They also refrained from caffeine since waking and from alcohol for the 24 h before each visit. Upon arrival at the laboratory, the participants completed the PSS and their baseline cognitive assessment. They then consumed their treatment for that day and, following a 60 min absorption period, they completed a second cognitive assessment. The testing protocol on Day 29 mirrored that of Day 1, with one exception: after completing the Day 1 assessments, the participants were provided with a treatment diary and the treatments that they would consume between the visits. On Day 29 the participants returned their remaining treatments and diary so that compliance could be calculated, and at the end of the session the participants were asked to complete a treatment guess form. Each testing visit had a duration of 2 h, as illustrated in Figure 3. Participants received £50 compensation upon the successful completion of the study, with pro-rata adjustments for participants who withdrew from the study, as requested. Testing visits were 28 days apart (mean 28.51 days; range 26–36 days).

### 2.5. Statistics

As shown in Figure 2, the data from the individual tasks were collapsed into an episodic memory cognitive domain (derived from the accuracy scores of each task relating to episodic memory converted to Z scores) and two global performance scores: global accuracy and global speed of performance (average of the accuracy or speed Z scores). The outcomes from the individual tasks shown in Figure 2 were also analysed as secondary outcomes.

Acute (Day 1) effects: In order to explore the acute effects of lion’s mane, data from Day 1 were entered into a two-way (treatment [lion’s mane, placebo] × timepoint [baseline, post-dose]) ANOVA.

Chronic (Day 29) effects: In order to explore the chronic effects of lion’s mane after 28 days of supplementation, data were entered into a two-way (treatment [lion’s mane, placebo] × timepoint [Day 1 baseline, Day 29 baseline]) ANOVA.

For all analyses, planned comparisons for each treatment group during each assessment were conducted. Only those planned comparisons relating to an outcome that generated a significant ANOVA result are reported.

## 3. Results

Due to the number of statistical analyses conducted, only the outcomes which revealed significant main or interaction effects, including treatment are reported below. Results of all statistical analyses can be found in the Supplementary Materials (see Tables S3–S8).

### 3.1. The Acute Effects of Lion’s Mane after a Single Dose (Day 1)

**Figure 4:**
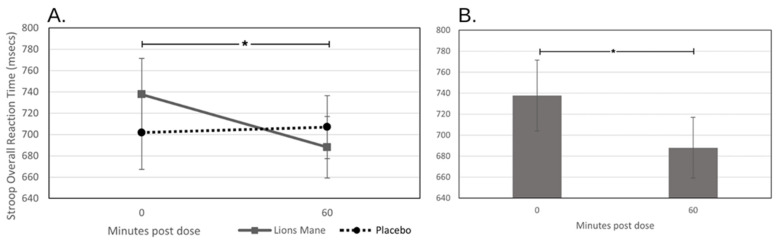
Acute effects on Stroop task reaction time. Effects of a single dose of lion’s mane on speed of performance on the Stroop task, showing the interaction effect (treatment × timepoint) (panel (**A**)) and the planned comparisons (panel (**B**)) conducted on the data from Day 1. * = *p* < 0.05.

There was a significant treatment × timepoint interaction with regard to the speed of performance on the Stroop task [*F* (1, 41) = 5.284, *p =* 0.027], with the participants performing significantly quicker following lion’s mane administration during the 1 h post-dose assessment [*F* (1, 41) = 8.897, *p* = 0.005]. The planned comparisons indicates that the lion’s mane participants performed quicker during the post-dose assessment (mean = 688.05 msecs) compared to the baseline assessment (mean = 737.70 msecs). See Figure 4. In contrast, a significant treatment × timepoint interaction was observed for the immediate word recall accuracy [*F* (1, 41) = 8.028, *p* = 0.007], where the participants recalled fewer correct responses at the post-dose assessment following lion’s mane administration [*F* (1, 41) = 6.744, *p* = 0.013]. Additionally, a significant treatment × timepoint interaction was observed for the immediate word recall errors [*F* (1, 41) = 5.831, *p* = 0.020], where the participants in the placebo condition performed fewer errors during the post-dose assessment [*F* (1, 41) = 11.396, *p* = 0.002]. There was also a significant interaction effect for the episodic memory factor, but the planned comparisons showed that there were no significant differences between the treatments during either assessment.

### 3.2. The Chronic Effects of Lion’s Mane 28-Day Treatment

**Figure 5:**
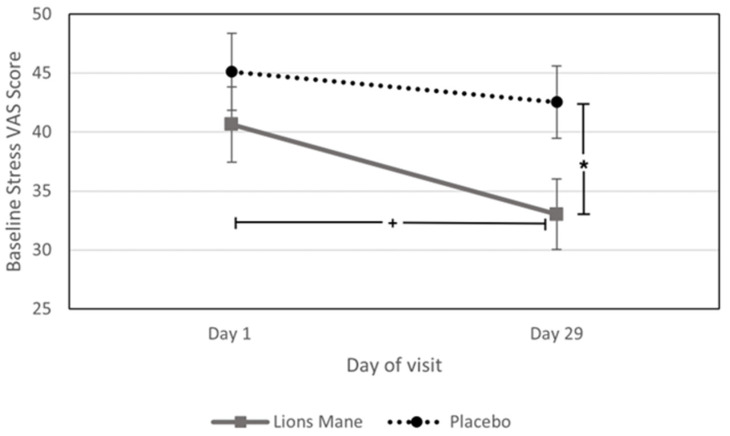
Chronic effects on response to Stress VAS. Effects of 28-day supplementation of lion’s mane on response to Stress VAS showing the trending main effect of treatment (*p* = 0.051). * = *p* < 0.05; + = *p* < 0.07.

A trend towards a significant chronic main effect of treatment was observed on Stress VAS [*F* (1, 39) = 4.059, *p =* 0.051], with the participants reporting significantly lower scores in subjective stress [*F* (1, 39) = 4.914, *p* = 0.033] on Day 29 following lion’s mane administration (mean = 33.02), in comparison to placebo (mean = 42.53). Additionally, on the same measure pairwise comparison indicated a trend towards a significant reduction in stress scores on Day 29 for the lion’s mane group (mean = 33.02) when compared to Day 1 scores (mean 40.64) [*F* (1, 39) = 3.913, *p* = 0.055]. See Figure 5.

In contrast, a significant treatment × timepoint interaction was observed for the delayed word recall accuracy [*F* (1, 39) = 5.318, *p* = 0.027], where participants in the placebo group recalled significantly more words at Day 29 in comparison to Day 1 [*F* (1, 39) = 14.669, *p* <0.001]. There was also a trend towards a significant interaction effect for the episodic memory factor, but the planned comparisons showed that there were no significant differences between treatments during either assessment.

## 4. Discussion

The current pilot trial explored the effects of *H. erinaceus* on cognitive function and mood in young, healthy adults. Expanding on the limited research to date, this trial assessed effects following a single 1.8 g dose of *H. erinaceus*, and again following 28 days of supplementation. The results of the trial indicated that following a single dose of *H. erinaceus,* participants performed significantly quicker on the Stroop task. Additionally, a potential stress-reducing effect was also observed following the 28 days of supplementation, with a trend towards reduction in subjective stress in the *H. erinaceus* group, both in comparison to the placebo and Day 1 scores.

When first considering the speed improvement, it must be highlighted that this was only observed on a singular task (Stroop) and was not seen for any other task nor the global speed of performance domain, as such this result should be taken with caution. Nevertheless, this is a novel finding to the area, where although previous enhancements to cognitive decline have been observed, no effects on speed of performance have been seen. This includes a previous trial in healthy, young adults, where no effects were observed on Stroop task performance when presented as a dual task challenge [23]. Therefore, this is the first study to demonstrate cognitive benefits of *H. erinaceus* in a young, healthy sample following a single dose of *H. erinaceus,* thus adding to the growing body of literature which has shown cognitive improvements following chronic supplementation in older adults with diagnosed mild cognitive impairment [20] and mild Alzheimer’s disease [21] and no cognitive impairments [22]. When considering mechanisms that may underlie this effect, it is well established that BDNF regulates synaptic plasticity and processes related to learning and memory consolidation [35]; however, there is less evidence pertaining to other areas of cognitive function. Recent work in older adults revealed an interaction between white matter hyperintensities, BDNF and processing speed [36], whereby those with low BDNF also had decreased processing speed. This suggests that the BDNF effects elicited by *H. erinaceus* may have the potential to improve processing speed, alongside learning and memory.

The current results also indicated a trend towards stress reduction, as measured using the Stress Visual Analogue Scales, following chronic consumption of *H. erinaceus*. Previous work has shown mood-enhancing effects following chronic administration of *H. erinaceus* [19,26], with both studies showing improvements in depression and anxiety scores. The preliminary evidence presented here also suggests there may be beneficial effects on subjective stress following *H. erinaceus* supplementation. Granted it must be recognized that this potential stress-alleviating effect was only observed on completion of the Stress Visual Analogue Scales and no difference was observed for treatment groups for responses to the Perceived Stress Scale. As detailed in a recent review, there are several mechanisms of action that might underpin the mood effects of *H. erinaceus* [37], namely that several bioactive compounds of *H. erinaceus* can stimulate the expression of neurotrophic factors including nerve growth factor [11,38,39,40]. Interestingly, serum nerve growth factor levels have previously been observed to be significantly reduced in individuals with major depressive disorder, compared to control samples [41,42]. This suggests that the ability of the bioactive compounds within *H. erinaceus* to interact with nerve growth factor may be crucial in producing mood-enhancing effects. Another mechanism of action is the potential modulatory abilities of *H. erinaceus* on monoamine neurotransmitters. Here, in restraint-stressed animals, chronic administration of 400 mg/kg *H. erinaceus* mycelium extract restored depleted expression levels of dopamine, serotonin and norepinephrine [43]. However, the modulation pathway here remains unknown and further investigation is required to determine how *H. erinaceus* could modulate monoamine neurotransmitters. Additionally, the anti-inflammatory effects of *H. erinaceus* may exert mood enhancing effects by modulation of the expression of IL-6, TNF-α and NF-κB [37,43]. Finally, given that BDNF at both basic mechanism level, and variants, is suggested as an arbitrator of vulnerability to stress and stress-related disorders [44], the ability of *H. erinaceus* to interact with BDNF pathways may offer a mechanistic explanation for mood-enhancing effects. Based on the previous work and the preliminary results presented here, alongside the mechanistic evidence, there is increasing indication that *H. erinaceus* can have mood-modulatory effects. The potential stress-reducing effects of chronic supplementation offer an interesting future avenue for intervention trials. Here, it would be interesting to investigate if *H. erinaceus* can exert physiological effects in response to stress, alongside subjective changes, for example, by subjecting the participants to an artificial laboratory stressor; this methodological approach has previously been sensitive in the response to other nutritional interventions [45,46,47].

Concerning the other measures, it must also be acknowledged that following acute administration of *H. erinaceus*, the participants performed less accurately on the immediate word recall, and participants who received the placebo performed better at the delayed word recall following chronic consumption. In addition, no treatment-related effects were observed on any other individual task outcome, nor on the cognitive domain global scores. Whilst null cognitive findings have previously been observed following *H. erinaceus* administration, the negative effect is difficult to explain, especially given that much of the previous work suggests a memory-enhancing effect following chronic intervention, although these are limited to animal work and cognitively compromised human cohorts. Whilst no differences in subjective alertness were observed here, it is tempting to suggest that the treatment-related differences could be attributed to differences in task engagement between the groups. A further consideration is that the composition of the placebo was responsible for memory enhancements, however standard-use microcrystalline cellulose was employed here, and therefore no active effects could be attributed to the composition of this treatment condition. The treatment guess confirmed that the blinding procedures were effective, where participants were not able to correctly identify which treatment they received; therefore, it is unlikely that any expectancy effects influenced cognitive performance. Further work is therefore necessary to better understand the effects of *H. erinaceus* on short-term memory in adults without cognitive compromise.

Given the nature of pilot trials, an inherent limitation of this trial is the small sample size utilized. This must be considered when considering the findings of this trial, as it was likely underpowered to detect the probable, modest cognition-enhancing effects in a healthy sample. Here, the demographic sample is likely at the peak of its cognitive abilities [48] and therefore any *H. erinaceus*-induced enhancements are expected to be very subtle. Previous trials have overcome this by incorporating methodological designs which subject healthy, young participants to highly demanding cognitive conditions (for example [29,33,49,50,51]). Therefore, it is probable that the cognitive assessment utilized within this trial was not demanding enough to detect the subtle behavioral changes. As such, future work in larger sample sizes should consider employing methodological designs which require the participant to sustain a high level of cognitive demand and induce mental fatigue. An additional consideration for the design of future trials is the timepoint for any assessment of acute effects. It may be that the 60 min timepoint utilized in this trial was too short to observe these effects, however, without human bioavailability data it is difficult to make this decision. Nonetheless, in a rat model following oral administration of *H. erinaceus* mycelia extract, plasma concentrations of erinacine S increased sharply and reached a peak concentration at 270 min post dose [10]; therefore, it may be more beneficial to employ additional post-dose assessments to better understand the acute effects of *H. erinaceus.*

## 5. Conclusions

In conclusion, this pilot trial tentatively suggests that *H. erinaceus* may improve speed of performance and exert a stress-reduction effect. However, it is crucial that further investigation is conducted employing larger sample sizes. Despite this, the key advantage of this trial is that the novel findings can be used in the development of supplementary trials to further investigate the effects of *H. erinaceus* on cognitive function, mood and wellbeing. The research field to date is still within its infancy, and findings from this trial offer a promising avenue of interest, and further work is encouraged in this area.

## Data Availability

The data presented in this study are available upon request from the corresponding author.

## Supplementary Materials

The following supporting information can be downloaded at: https://www.mdpi.com/article/10.3390/nu15224842/s1, Table S1: Participant characteristics at enrolment for continuous variables. *n* = 43; Table S2: Participant characteristics at enrolment for categorical variables. *n* = 43. Table S3: Acute performance on the global cognitive performance scores on Day 1. Table S4: Chronic performance on the global cognitive performance scores on Day 29. Table S5: Acute performance on the COMPASS cognitive task individual outcomes on Day 1. Table S6: Chronic performance on the COMPASS cognitive task individual outcomes on Day 29. Table S7: Acute performance on mood outcomes on Day 1. Table S8: Chronic performance on mood outcomes on Day 29.
