# Supplementary material for: The Acute and Chronic Effects of Lion’s Mane Mushroom Supplementation on Cognitive Function, Stress and Mood in Young Adults: A Double-Blind, Parallel Groups, Pilot Study
Source: Nutrients. 2023 Nov 20;15(22):4842. doi: 10.3390/nu15224842 (PMC10675414; doi:10.3390/nu15224842)
Supplement: Supplementary file 1 [file nutrients-15-04842-s001.zip › nutrients-2705549-supplementary.pdf]

Supplementary Materials

1. Eligibility Criteria

Inclusion criteria

- Participants are aged 18-45 years, inclusive
- Participants self-report that they are in good health
- Participants are a native speaker of English or fluent in English (equivalent to IELTS Level 6)
- Understanding the study procedures and willing to provide informed consent to participate in the study

Exclusion criteria

Participants complying with at least one of the following criteria were not eligible:

- Failure to meet any one of the inclusion criteria
- Current use of prescription medication, with some specific exceptions including contraceptives, medications used in the treatment of hypo/hyperthyroidism and as required in the treatment of hayfever and mild asthma
- Have a pre-existing diagnosed medical condition/illness, with the explicit exception of controlled hypo/hyperthyroidism, mild asthma and hayfever
- Have a diagnosed neurological condition or assessed as having a learning/behavioural or neurodevelopmental difference such as dyslexia or ADHD
- Have a visual impairment that cannot be corrected with glasses or contact lenses, including colour blindness
- Food allergies/intolerances/sensitivities to any ingredients in the study products
- Are pregnant, trying to get pregnant or lactating
- Smoke tobacco, vape nicotine or use nicotine replacement products
- Have taken antibiotics within the past 4 weeks
- Have taken dietary supplements e.g. Vitamins, omega 3 fish oils etc. in the last 4 weeks (Note: participation is possible following a 4 week supplement washout prior to participating and for the duration of the study on the proviso that the supplements they are taking are out of choice and not medically prescribed or advised) With the exception here of vitamin D and iron supplements, where they have been medically advised and have been taken consistently for a minimum of 4 weeks and will be taken consistently throughout the trial
- Have any health condition that would prevent fulfilment of the study requirements (this includes non-diagnosed conditions for which no medication may be taken)
- Unable to demonstrate adequate minimal performance on lab, computer-based cognitive tasks
- Has been diagnosed with/ undergoing treatment for alcohol or drug abuse in the last 12 months
- Have been diagnosed with/ undergoing treatment for a psychiatric disorder in the last 12 months
- Excessive caffeine intake (>300 mg per day)
- Have a Body Mass Index (BMI) outside of the range 18.5-35 kg/m^2

2. Participant characteristics at enrolment

Table S1. Participant characteristics at enrolment for continuous variables. *n* = 43

Measure	Mean	SD
Age (years)	26.35	6.12
Education (years)	17.12	2.48
Fruit & veg consumption (portions/day)	3.03	1.59
Caffeine consumption (mg/day)	85.92	73.65
Systolic BP (mm/Hg)	120.53	12.62
Diastolic BP (mm/Hg)	76.82	8.68
Heart Rate (beats per minute)	74.45	13.64
BMI (kg/m^2)	24.02	3.42
WHR	.85	.08

Table S2. Participant characteristics at enrolment for categorical variables. *n* = 43

Measure	Count	%
Male/female	20/23	46.5/53.5
Race:		
White	29	67.4
Black	3	6.9
Asian	6	13.9
Other	5	11.6
Wear glasses (yes/no)	6/37	14/86
Handedness (left/right)	0/43	0/100
Dietary habits:		
Omnivore	38	88.4
Vegetarian	3	7
Pescatarian	1	2.3
Other	1	2.3
Highest level of education:		
Entry Level (Entry Level Certificates)	0	0.0
Level 1 (NVQ level 1, GCSE D-G, BTEC Intro)	0	0.0
Level 2 (NVQ level 2, GCSE A*-C, Young Apprenticeships, BTEC ½)	1	2.3
Level 3 (NVQ level 3, AS & A-levels, BTEC 3)	12	27.9
Level 4 (NVQ level 4, Cert of Higher Ed, BTEC 4)	1	2.3
Level 5 (NVQ level 5, HND, HNC, Dip HE/FE, Found. Deg, BTEC 5)	2	4.7
Level 6 (Bachelors Degree, GradDip, Grad Cert., BTEC 6)	15	34.9
Level 7 (Masters, PGDip, PG Cert, BTEC 7)	9	20.9
Level 8 (Doctorates, BTEC 8)	3	7

3. Computerised cognitive tasks

Word Presentation: A unique set of fifteen words will be presented. Words will be selected at random from a large bank of words (MRC Psycholinguistic Database) matched for word length, frequency, familiarity and concreteness. Stimulus duration will be one second, as will be the inter-stimulus duration.

Immediate and Delayed Word Recall: Participants write down as many of the 15 words that they were presented during the stimulus presentation period at two time points: immediately and at the end of the cognitive assessment (delayed) This task is scored for accuracy only.

Numeric Working Memory: a series of numbers are displayed on the screen, one at a time. Participants are required to memorize these numbers as they appear. Once the series is complete, numbers are displayed one at a time and participants are required to indicate if each number was presented in the previous list or not.

Choice Reaction Time: an arrow appears on the screen pointing to the left or to the right. Participants respond with a left or right key press corresponding to the direction of the arrow. There is a randomly varying inter-stimulus interval of between 1 and 3 seconds for a total of fifty stimuli.

Stroop: words describing one of four colours ('RED', 'YELLOW', 'GREEN', 'BLUE') are presented in different coloured fonts on screen. In this case 60 'congruent' (word and font are the same colour) or 'incongruent' (word and font are different colours) were presented in a random order.

Peg and Ball: Two configurations are shown on the screen each containing three coloured balls on one of three pegs. Participants must arrange the balls on the starting configuration to match the position of balls in the goal configuration in the least number of moves possible

Delayed Word Recognition: A series of words are displayed on the screen, one at a time. The number of pictures, the rate at which they are displayed and the interstimulus interval can be modified. All target words shown during Word Presentation plus an equal number of decoys will be displayed on the screen one at a time. For each stimulus participants select 'Yes' or 'No' to indicate if they have seen the word before or not. The task outcomes include accuracy and reaction time.

4. Response to intervention

Table S3. Acute Performance on the global cognitive performance scores on Day 1.

Baseline Z scores (plus standard deviation) were collected prior to dosing. Data presented from 60 minutes post dose (p.d.) assessment are Z scores (plus standard deviation). Final two columns showing test statistics (F) and associated probabilities (*p*).

			Baseline		60 min p.d.				
		N	Mean	SD	Mean	SD		F	*p*
Day 1									
Speed of Performance	LMM	22	0.118	0.860	0.063	0.761	Treatment	0.621	0.435
	PLA	21	-0.124	-0.713	-0.066	0.798	Treat*Time	1.639	0.208
Accuracy of Performance	LMM	22	0.048	0.652	-0.006	0.511	Treatment	0.075	0.786
	PLA	21	-0.050	0.493	0.007	0.532	Treat*Time	0.694	0.410
Episodic Memory	LMM	22	0.212	0.810	-0.050	0.885	Treatment	0.506	0.481
	PLA	21	-0.222	0.866	0.052	0.848	Treat*Time	5.343	0.026

Table S4. Chronic performance on the global cognitive performance scores on Day 29.

Baseline Z scores (plus standard deviation) were collected prior to dosing at Day 1. Data presented Day 29 are Z scores (plus standard deviation) collected prior to dosing at Day 29. Final two columns showing test statistics (F) and associated probabilities (*p*).

			Day 1 (V1A1)		Day 29 (V2A1)				
		N	Mean	SD	Mean	SD		F	*p*
Speed of Performance	LMM	21	0.069	0.850	0.116	0.906	Treatment	0.875	0.355
	PLA	20	-0.146	0.724	-0.122	0.628	Treat*Time	0.063	0.803
Accuracy of Performance	LMM	21	0.014	0.649	-0.046	0.609	Treatment	0.013	0.910
	PLA	20	-0.043	0.505	0.048	0.528	Treat*Time	0.907	0.347
Episodic Memory	LMM	21	0.190	0.823	-0.037	0.694	Treatment	0.420	0.567
	PLA	20	-0.173	0.858	0.039	1.106	Treat*Time	3.396	0.073

Table S5. Acute performance on the COMPASS cognitive task individual outcomes on Day 1.

The Baseline data are raw means (plus SD) collected prior to dosing. Data presented from the 60 post dose (p.d.) assessments are raw means (plus SD). With the final two columns showing test statistics (F) and associated probabilities (*p*).

			Baseline		60 min p.d.				
		N	Mean	SD	Mean	SD		F	*p*
					Day 1				
Numeric Working Memory – Accuracy (%)	LMM	22	96.314	3.636	96.112	3.476	Treatment	0.349	0.558
	PLA	21	97.621	2.173	95.769	3.214	Treat*Time	2.431	0.127
Numeric Working Memory – Overall RT (Msecs)	LMM	22	861.647	339.566	833.566	256.784	Treatment	0.346	0.559
	PLA	21	802.521	213.750	800.876	229.697	Treat*Time	0.371	0.546
Choice Reaction Time – Accuracy (%)	LMM	22	97.64	3.185	98.55	1.654	Treatment	0.173	0.679
	PLA	21	98.00	2.898	97.62	2.334	Treat*Time	2.521	0.120
Choice Reaction Time – Overall RT (Msecs)	LMM	22	470.004	112.248	468.381	90.866	Treatment	0.239	0.628
	PLA	21	436.988	99.999	465.968	181.609	Treat*Time	1.471	0.232
Stroop – Accuracy (%)	LMM	22	96.438	5.099	96.061	4.380	Treatment	0.725	0.400
	PLA	21	97.142	3.254	97.221	2.317	Treat*Time	0.215	0.645
Stroop – Overall RT (Msecs)	LMM	22	737.669	164.409	688.053	119.153	Treatment	0.038	0.846
	PLA	21	701.832	152.272	706.931	150.675	Treat*Time	5.284	0.027
Peg and Ball – Thinking RT (Msecs)	LMM	22	2657.32	1795.329	2765.82	2151.912	Treatment	1.657	0.205
	PLA	21	2123.33	995.321	2066.86	1126.737	Treat*Time	0.741	0.394
Peg and Ball – Complete RT (Msecs)	LMM	22	7989.36	2093.397	7719.68	2437.220	Treatment	1.357	0.251
	PLA	21	7341.05	2560.635	6840.14	1773.819	Treat*Time	0.367	0.548
Peg and Ball – Errors	LMM	22	3.45	3.609	2.91	2.653	Treatment	0.328	0.570
	PLA	21	3.14	4.486	2.33	2.763	Treat*Time	0.034	0.854
Word Recognition – Accuracy (%)	LMM	22	82.880	10.199	80.757	9.081	Treatment	0.494	0.486
	PLA	21	79.048	8.953	81.270	8.529	Treat*Time	2.013	0.164

Word Recognition – Overall RT (Msecs)	LMM	21	967.088	323.026	1005.603	342.424	Treatment	0.569	0.455
	PLA	22	920.599	198.277	923.118	267.274	Treat*Time	0.660	0.421
Immediate Word Recall - Correct	LMM	22	7.795	2.169	6.523	2.270	Treatment	0.004	0.950
	PLA	21	6.762	2.391	7.476	2.615	Treat*Time	8.028	0.007
Immediate Word Recall - Errors	LMM	22	0.50	1.012	0.50	0.673	Treatment	0.171	0.681
	PLA	21	0.90	0.995	0.29	0.717	Treat*Time	5.831	0.020
Delayed Word Recall - Correct	LMM	22	5.545	2.595	4.636	3.270	Treatment	1.184	0.283
	PLA	21	4.357	2.511	4.214	2.813	Treat*Time	0.770	0.385
Delayed Word Recall – Errors	LMM	22	1.00	1.345	0.86	1.424	Treatment	0.021	0.886
	PLA	21	1.14	1.389	0.62	1.244	Treat*Time	0.828	0.368

Table S6. Chronic performance on the COMPASS cognitive task individual outcomes on Day 29.

All data show raw means (plus SD) collected prior to dosing at Day 1 and Day 29. Final two columns showing test statistics (F) and associated probabilities (*p*).

			Baseline (V1A1)		Day 29 (V2A1)				
		N	Mean	SD	Mean	SD		F	*p*
Numeric Working Memory – Accuracy (%)	LMM	21	96.138	3.629	95.662	4.387	Treatment	0.947	0.337
	PLA	20	97.502	2.159	96.002	3.131	Treat*Time	0.675	0.416
Numeric Working Memory – Overall RT (Msecs)	LMM	21	860.524	347.910	810.236	265.924	Treatment	0.408	0.527
	PLA	20	795.697	216.944	774.441	181.698	Treat*Time	0.424	0.519
Choice Reaction Time – Accuracy (%)	LMM	21	97.52	3.219	97.81	6.063	Treatment	0.000	0.988
	PLA	20	97.90	2.936	97.40	2.257	Treat*Time	0.512	0.478
Choice Reaction Time – Overall RT (Msecs)	LMM	21	470.824	114.952	548.239	333.118	Treatment	1.246	0.271
	PLA	20	435.107	102.216	460.363	152.478	Treat*Time	0.805	0.375
Stroop – Accuracy (%)	LMM	21	96.269	5.161	94.603	10.191	Treatment	1.012	0.321
	PLA	20	97.000	3.271	97.166	3.111	Treat*Time	0.784	0.381
Stroop – Overall RT (Msecs)	LMM	21	737.910	168.465	783.046	240.068	Treatment	0.931	0.341
	PLA	20	699.352	155.793	714.166	160.167	Treat*Time	0.957	0.334
Peg and Ball – Thinking RT (Msecs)	LMM	21	2488.95	1652.135	2393.19	1784.925	Treatment	0.858	0.360
	PLA	20	2093.60	1011.563	2012.55	892.663	Treat*Time	0.003	0.854
Peg and Ball – Complete RT (Msecs)	LMM	21	7761.24	1843.712	7651.19	2282.444	Treatment	0.808	0.374
	PLA	20	7342.60	2627.146	6882.95	2047.563	Treat*Time	0.704	0.407
Peg and Ball – Errors	LMM	21	3.62	3.612	2.67	2.129	Treatment	0.392	0.535
	PLA	20	3.15	4.603	2.25	1.803	Treat*Time	0.001	0.971
Word Recognition – Accuracy (%)	LMM	21	82.382	10.173	81.270	5.722	Treatment	0.498	0.485
	PLA	20	79.334	9.087	81.334	8.475	Treat*Time	0.927	0.341
	LMM	21	925.849	265.101	911.531	265.546	Treatment	0.11	0.741

Word Recognition – Overall RT (Msecs)	PLA	20	904.096	188.048	886.273	219.516	Treat*Time	0.006	0.940
Immediate Word Recall - Correct	LMM	21	7.738	2.206	7.690	1.820	Treatment	0.318	0.576
	PLA	20	6.950	2.288	7.750	2.557	Treat*Time	2.679	0.110
Immediate Word Recall - Errors	LMM	21	0.52	1.030	0.19	0.402	Treatment	1.985	0.167
	PLA	20	0.80	0.894	0.40	0.681	Treat*Time	0.036	0.851
Delayed Word Recall - Correct	LMM	21	5.571	2.657	5.881	2.334	Treatment	0.160	0.691
	PLA	20	4.450	2.539	6.400	3.081	Treat*Time	5.318	0.027
Delayed Word Recall – Errors	LMM	21	1.05	1.359	0.38	0.498	Treatment	0.022	0.883
	PLA	20	1.05	1.356	0.45	0.759	Treat*Time	0.021	0.885

Table S7. Acute performance on mood outcomes on Day 1.

Data derived from scores on the Visual Analogue Mood Scales (VAMS) and Stress Visual Analogue Scales (S-VAS). The Baseline data are raw means (plus SD) collected prior to dosing. Data presented from the 60 post dose (p.d.) assessments are raw means (plus SD). With the final two columns showing test statistics (F) and associated probabilities (*p*).

			Baseline		60 min p.d.				
		N	Mean	SD	Mean	SD		F	*p*
Day 1									
Alertness (VAMS)	LMM	22	58.421	13.366	59.864	10.745	Treatment	0.076	0.784
	PLA	21	57.035	12.845	59.169	14.120	Treat*Time	0.113	0.738
Stress (VAMS)	LMM	22	42.580	9.650	40.682	12.062	Treatment	1.305	0.260
	PLA	21	47.202	10.435	43.726	15.383	Treat*Time	0.275	0.603
Tranquillity (VAMS)	LMM	22	63.545	13.823	64.909	10.225	Treatment	0.828	0.368
	PLA	21	60.238	13.669	61.111	16.273	Treat*Time	0.029	0.865
Stress Score (VAS)	LMM	22	40.011	14.698	40.374	9.982	Treatment	1.117	0.297
	PLA	21	44.798	14.190	43.012	12.553	Treat*Time	0.346	0.560

Table S8. Chronic performance on mood outcomes on Day 29.

Data derived from scores on the Visual Analogue Mood Scales (VAMS), Stress Visual Analogue Scales (S-VAS) and Perceived Stress Scale (PSS) at Day Day 29. All data show raw means (plus SD) collected prior to dosing at Day 1 and Day 29. Final two columns showing test statistics (F) and associated probabilities (*p*).

			Baseline (V1A1)		Day 29 (V2A1)				
		N	Mean	SD	Mean	SD		F	*p*
Alertness (VAMS)	LMM	21	57.385	12.759	60.723	11.660	Treatment	0.066	0.799
	PLA	20	57.386	13.075	58.777	16.064	Treat*Time	0.281	0.599
Stress (VAMS)	LMM	21	42.464	9.871	39.536	10.865	Treatment	1.154	0.289
	PLA	20	47.025	10.674	41.700	15.559	Treat*Time	0.353	0.556
Tranquillity (VAMS)	LMM	21	62.921	13.842	66.270	10.295	Treatment	0.870	0.357
	PLA	20	60.200	14.023	61.700	16.482	Treat*Time	0.258	0.614
Stress Score (VAS)	LMM	21	40.643	14.752	33.024	12.585	Treatment	4.059	0.051
	PLA	20	45.113	14.483	42.538	14.852	Treat*Time	0.837	0.366
PSS	LMM	21	15.590	5.279	15.285	4.051	Treatment	0.000	0.998
	PLA	20	17.000	7.141	15.750	5.437			
